# Supplementary material for: Formicine ants swallow their highly acidic poison for gut microbial selection and control
Source: eLife. 2020 Nov 3;9:e60287. doi: 10.7554/eLife.60287 (PMC7609056; doi:10.7554/eLife.60287)
Supplement: Transparent reporting form [file elife-60287-transrepform.docx]

***eLife’s* transparent reporting form**

We encourage authors to provide detailed information *within their submission* to facilitate the interpretation and replication of experiments. Authors can upload supporting documentation to indicate the use of appropriate reporting guidelines for health-related research (see [EQUATOR Network](http://www.equator-network.org/%20)), life science research (see the [BioSharing Information Resource](https://biosharing.org/)), or the [ARRIVE guidelines](http://www.plosbiology.org/article/info:doi/10.1371/journal.pbio.1000412) for reporting work involving animal research. Where applicable, authors should refer to any relevant reporting standards documents in this form.

If you have any questions, please consult our Journal Policies and/or contact us: [editorial@elifesciences.org](mailto:editorial@elifesciences.org).

**Sample-size estimation**

- You should state whether an appropriate sample size was computed when the study was being designed
- You should state the statistical method of sample size computation and any required assumptions
- If no explicit power analysis was used, you should describe how you decided what sample (replicate) size (number) to use

Please outline where this information can be found within the submission (e.g., sections or figure legends), or explain why this information doesn’t apply to your submission:

No statistical methods were used to pre-determine sample size. Adequate sample sizes were determined through experience gained in previous studies investigating the use of poison gland secretions as topical disinfectant:

-Tragust, S. et al. 2013 Ants disinfect fungus-exposed brood by oral uptake and spread of their poison. Curr Biol 23, 76-82.

-Pull, C. D. et al. 2018 Destructive disinfection of infected brood prevents systemic disease spread in ant colonies. eLife 7, e32073.

Sample sizes, including number of workers per ant colony as well as number of ant colonies, are reported in the materials and methods section.

**Replicates**

- You should report how often each experiment was performed
- You should include a definition of biological versus technical replication
- The data obtained should be provided and sufficient information should be provided to indicate the number of independent biological and/or technical replicates
- If you encountered any outliers, you should describe how these were handled
- Criteria for exclusion/inclusion of data should be clearly stated
- High-throughput sequence data should be uploaded before submission, with a private link for reviewers provided (these are available from both GEO and ArrayExpress)

Please outline where this information can be found within the submission (e.g., sections or figure legends), or explain why this information doesn’t apply to your submission:

All experimental findings reported in this study are based on replicated data (biological replicates) obtained from several colonies of the same ant species or from colony fragments of several different ant species.

In the analyses of count data on the number of bacteria in vivo and in vitro (Fig. 2, Fig. 5 and Fig. 5 – figure supplement 2), we removed predictor levels with all zeros, as inclusion of these levels in statistical models results in complete data separation, i.e. the predictor variable perfectly predicts the outcome, with maximum likelihood estimates lying on the boundary of the parameter space. Data omitted in the analyses is however explicitly shown in the figures and reported in the text and the figure legends. This information is also clearly reported in the section material and methods.

**Statistical reporting**

- Statistical analysis methods should be described and justified
- Raw data should be presented in figures whenever informative to do so (typically when N per group is less than 10)
- For each experiment, you should identify the statistical tests used, exact values of N, definitions of center, methods of multiple test correction, and dispersion and precision measures (e.g., mean, median, SD, SEM, confidence intervals; and, for the major substantive results, a measure of effect size (e.g., Pearson's r, Cohen's d)
- Report exact p-values wherever possible alongside the summary statistics and 95% confidence intervals. These should be reported for all key questions and not only when the p-value is less than 0.05.

Please outline where this information can be found within the submission (e.g., sections or figure legends), or explain why this information doesn’t apply to your submission:

Statistical analysis of the data presented in the study is thoroughly described in the material and methods section, including sample sizes and statistical tests or models used together with a description of predictors and methods of multiple test correction. Dispersion and precision measures are given throughout the result section and exact p-values are reported down to three decimal places. All figures show raw data points alongside summarizing measures of center and dispersion.

(For large datasets, or papers with a very large number of statistical tests, you may upload a single table file with tests, Ns, etc., with reference to sections in the manuscript.)

**Group allocation**

- Indicate how samples were allocated into experimental groups (in the case of clinical studies, please specify allocation to treatment method); if randomization was used, please also state if restricted randomization was applied
- Indicate if masking was used during group allocation, data collection and/or data analysis

Please outline where this information can be found within the submission (e.g., sections or figure legends), or explain why this information doesn’t apply to your submission:

Allocation of ants into experimental groups was completely random and replicates of all groups in one experiment were set up at the same time. Investigators were not blinded to group allocation during data collection and analysis. To ensure unbiased results the following steps were undertaken:

1. different investigators performed experiments building upon each other, e.g. survival of individual ants and survival of donor-receiver ant pairs

2. different investigators performed experiments and analyzed data, e.g. bacterial growth in vivo

3. A different methodology was used to manipulate ants, e.g. survival of individual ants and survival of donor-receiver ant pairs, or to take a measurement, e.g. crop lumen acidification.

This information is clearly reported in the section material and methods.

**Additional data files (“source data”)**

- We encourage you to upload relevant additional data files, such as numerical data that are represented as a graph in a figure, or as a summary table
- Where provided, these should be in the most useful format, and they can be uploaded as “Source data” files linked to a main figure or table
- Include model definition files including the full list of parameters used
- Include code used for data analysis (e.g., R, MatLab)
- Avoid stating that data files are “available upon request”

Please indicate the figures or tables for which source data files have been provided:

All data supporting the findings of this study and all code required to reproduce the analyses and figures of this study have been be uploaded as additional data files and made publicly available at Dryad (http://doi.org/10.5061/dryad.k0p2ngf4v).
